# Supplementary material for: Effect of Cationic Lipid Nanoparticle Loaded siRNA with Stearylamine against Chikungunya Virus
Source: Molecules. 2022 Feb 9;27(4):1170. doi: 10.3390/molecules27041170 (PMC8877324; doi:10.3390/molecules27041170)
Supplement: Supplementary file 1 [file molecules-27-01170-s001.zip › molecules-1535050-supplementary.pdf]

Supplementary Materials

Effect of Cationic Lipid Nanoparticle Loaded siRNA with Stearylamine against Chikungunya Virus

Manish Kumar Jeengar [1,2,†], Mallesh Kurakula [3,4,†], Poonam Patil [1], Ashwini More [1], Ramakrishna Sistla [3,*] and Deepti Parashar [1,*]

1 ICMR-National Institute of Virology, 20-A, Dr. Ambedkar Road, Pune 411001, Maharashtra, India; manishkumarj@pharmacy.aims.amrita.edu (M.K.J.); poonamshewale07@rediffmail.com (P.P.); ashwini05.s@gmail.com (A.M.)
2 Department of Pharmacology, Amrita School of Pharmacy, Amrita Vishwa Vidyapeetham, AIMS Health Sciences Campus, Kochi 682041, Kerala, India
3 CSIR-Indian Institute of Chemical Technology [CSIR-IICT], Hyderabad 500007, Telangana, India; mallesh_kurakula@yahoo.com
4 Product Development, CURE Pharmaceutical, Oxnard, CA 93033, USA.
* Correspondence: sistla@csiriict.in (R.S.); deeptiparasharster@gmail.com (D.P.)
† These authors contributed equally to this work.

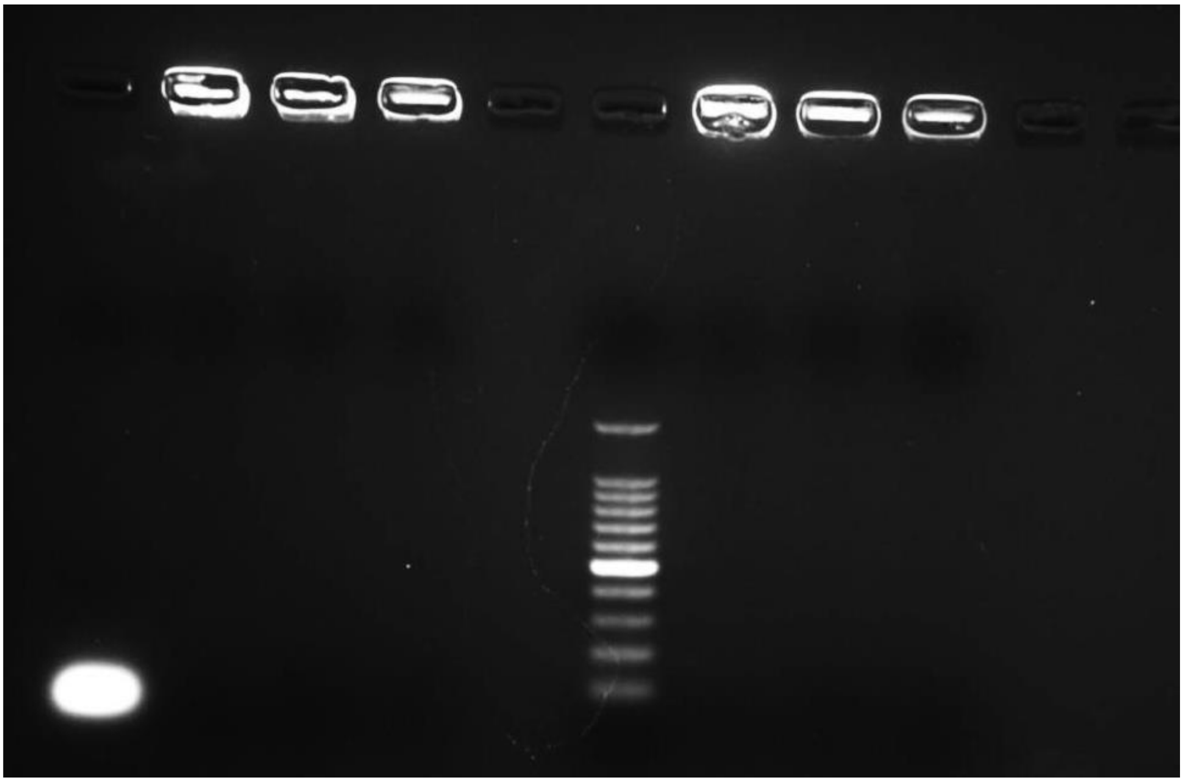

Figure S1. Agarose Gel electrophoresis results shows formation of Stable complexation of siRNA—F4 SLN; Lane 1 Naked siRNA; Lane 2–4 siRNA complexed with cationic SLN incubated at 4 °C; Lane 5 Blank SLN; Lane 6 DNA ladder; Lane 7–9, siRNA complexed with cationic SLN incubated at 37 °C.
